# The Prevalence and Genetic Variants of the CCHF Virus Circulating among Ticks in the Southern Regions of Kazakhstan

**DOI:** 10.3390/pathogens11080841

**Published:** 2022-07-27

**Authors:** Kulyaisan T. Sultankulova, Gaukhar O. Shynybekova, Nurlan S. Kozhabergenov, Nazym N. Mukhami, Olga V. Chervyakova, Yerbol D. Burashev, Kunsulu D. Zakarya, Aziz K. Nakhanov, Kainar B. Barakbayev, Mukhit B. Orynbayev

**Affiliations:** Research Institute for Biological Safety Problems of the Ministry of Health of the Republic of Kazakhstan, Gvardeiskiy 080409, Kazakhstan; gaukhar_1988@bk.ru (G.O.S.); nurlanks@gmail.com (N.S.K.); mukhami.nazym@mail.ru (N.N.M.); ovch@mail.ru (O.V.C.); mario_bur@mail.ru (Y.D.B.); rkm_kz@mail.ru (K.D.Z.); aziz_nk@mail.ru (A.K.N.); kainar7@mail.ru (K.B.B.)

**Keywords:** Crimean–Congo hemorrhagic fever, virus, tick, *Ixodes ricinus*, *Hyalomma marginatum*, *Hyalomma anatolicum*, *Hyalomma asiaticum*, *Hyalomma scupense*

## Abstract

Crimean–Congo hemorrhagic fever (CCHF) disease cases are registered annually in endemic regions of Kazakhstan. To study the prevalence of various Crimean–Congo hemorrhagic fever virus (CCHFV) genotypes, a total of 694 ticks were collected from southern regions of Kazakhstan in 2021. *Hyalomma marginatum* (*n* = 323) (46.5%), *Hyalomma anatolicum* (*n* = 138) (19.9%), *Hyalomma asiaticum* (*n* = 126) (18.2%), *Hyalomma scupense* (*n* = 80) (11.5%) and *Ixodes ricinus* (*n* = 27) (3.9%) were collected using the standardized flagging technique from the environment. All the tick samples were analyzed for the presence of CCHFV RNA by RT-PCR. The CCHF-positive samples were found within three *Hyalomma asiaticum* and one *Ixodes ricinus* tick sample. For the first time in Kazakhstan, infection of the *Ixodes ricinus* tick with CCHFV was detected. The results of sequencing and analysis of the S-gene fragment showed that the Asia 1 and Asia 2 CCHF genotypes circulate in the southern regions of Kazakhstan. Viruses isolated in the Zhambyl and Turkestan regions are assigned to the Asia-2 genotype, whereas the virus isolated in the Kyzylorda region to the Asia-1 genotype.

## 1. Introduction

CCHF is an acute transmissible natural focal viral infection characterized by fever, general intoxication and hemorrhagic symptom. The infection has a wide array of clinical manifestations, ranging from asymptomatic carrier and nasopharyngitis to meningitis, meningococcemia and meningococcal sepsis. Mortality rates can range from 5% to 30% [1,2].

The causative agent of the CCHF is a pathogenic virus belonging to the order *Bunyavirales*, family *Nairoviridae*, genus *Orthonairovirus* [3]. CCHFV is widely distributed in Europe, Asia and Africa, where its circulation has been detected in almost 40 countries [4,5,6]. It is mainly transmitted by ixodes ticks of the genus *Hyalomma*, and the distribution area of the virus almost completely coincides with the area where the ticks are most abundant [4]. In natural foci, the main feeders of ticks at different stages of their lifecycle are wild and domestic vertebrates: cattle and small cattle, hares, hedgehogs, jerboa and other small mammals. Birds serve as the feeders of preimaginal phases and can spread the pathogen over long distances [7,8]. CCHFV is one of the most genetically diverse representatives of the family *Nairovirus*. The virus genome is composed of single-stranded negative-sense RNA, which consists of three segments: small (S), medium (M) and large (L) [9,10]. Based on partial and complete nucleotide sequences of the S and L segments of the CCHFV genome, seven virus genotypes are distinguished: Africa-1, Africa-2, Africa-3, Asia-1, Asia-2, Euro-1 and Euro-2 [11,12]. CCHF has been registered in Kazakhstan since 1948. The active natural foci of CCHF on the territory of Kazakhstan are located in Zhambyl, South Kazakhstan and Kyzylorda regions [13]. In Kazakhstan, the first CCHF cases were reported in the Turkestan region in 1948 and received the name “Central Asian fever” [14,15]. Later, in 1964, cases of CCHF were registered in the Kyzylorda region [16] and in 1982 in the Zhambyl region [17]. The CCHF epidemic situation in natural foci remains unstable every year. Natural foci are gradually expanding their borders, especially intensively in Kyzylorda and South Kazakhstan regions. In Kazakhstan, about 16 annual clinical cases are registered [18], and the average mortality rate is 14.8% [19].

The first genetic analysis of CCHFV recovered in the southern part of Kazakhstan showed their genetic diversity. The authors established that the CCHFV isolated in the South Kazakhstan region belongs to the genetic group Asia 1 and Asia 2, which also included CCHFV strains from Kazakhstan, Uzbekistan, Tajikistan and China [20,21].

The purpose of this study was to determine the prevalence of the CCHFV among ticks in endemic areas of Kazakhstan and to identify their genetic diversity.

## 2. Results

### 2.1. Isolation of CCHFV from Ticks in the Southern Region of Kazakhstan

During the spring and summer of 2021, 694 ticks were collected in 3 endemic areas for CCHF, 209 from the Zhambyl region, 203 from the Turkestan region and 282 from the Kyzylorda region. Analysis of species composition showed that the composition of the collected adult ticks belongs to five different species: *Hyalomma marginatum* (*n* = 323) (46.5%), *Hyalomma anatolicum* (*n* = 138) (19.9%), *Hyalomma asiaticum* (*n* = 126) (18.2%), *Hyalomma scupense* (*n* = 80) (11.5%) and *Ixodes ricinus* (*n* = 27) (3.9%).

CCHFV RNA has been detected in 4 (0.57%) out of 694 adult ticks tested. One *Hyalomma asiaticum* (4.5% positive samples) in the Zhambyl region, two *Hyalomma asiaticum* (8.0%) in the Turkestan region and one *Ixodes ricinus* in the Kyzylorda region were infected with CCHFV (Table 1). The first case of CCHF viral infection by *Ixodes ricinus* on the territory of Kazakhstan has been established.

### 2.2. Determination of the Genotype of the CCHFV

**Figure 1:**
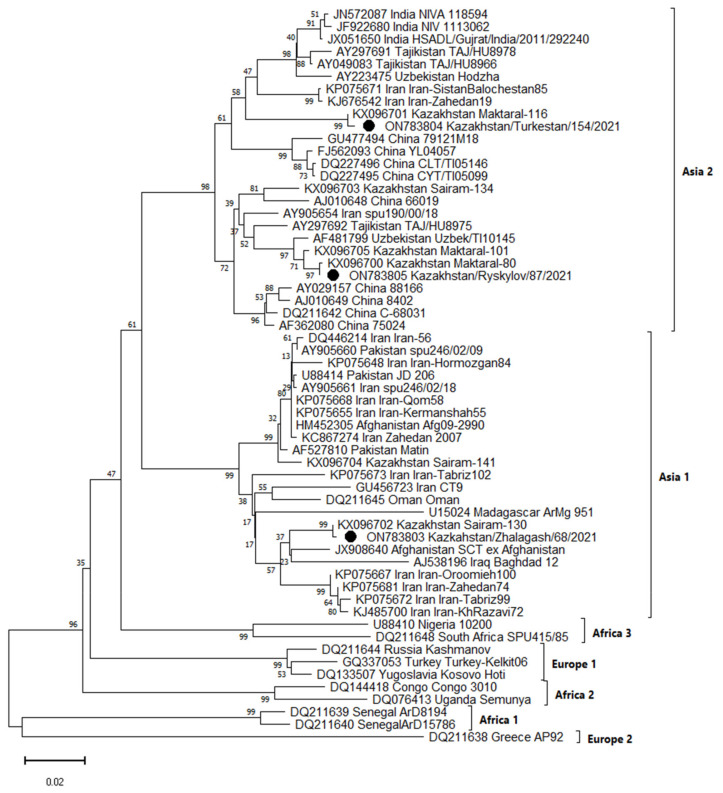
Phylogenetic tree based on the S-segment of the CCHFV genome. CCHFV strains isolated from ticks in Kazakhstan in 2021, belonging to the Asian lineage Asia 1 and Asia 2, are circled.

Sequencing of the S segment fragment and phylogenetic analysis of PCR-positive samples allowed us to cluster the obtained sequences into two groups belonging to the Asia 1 and Asia 2 genetic lineage (Figure 1).

The Kazakhstan/Ryskylov/87/2021 (ON783805) virus isolated in the Zhambyl region and the Kazakhstan/Turkestan/154/2021 (ON783804) virus isolated in the Turkestan region are similar in nucleotide sequence to viruses isolated in Kazakhstan (KX096701), (KX096705), (KX096703), (KX096700), Iran (KP075671), (KJ676542) Tajikistan (AY297692), Uzbekistan (AF481799) and China (AJ010648) and were clustered in the Asia 2 lineage. The Kazakhstan/Zhalagash/68/2021 (ON783803) virus isolated from ticks in the Kyzylorda region was identical to viruses from Kazakhstan (KX096702), (KX096704), Afghanistan (JX908640), Iraq (AJ538196) and Iran (KP075667) and belongs to the Asia 1 lineage.

Kazakh strains of CCHFV (KX096701, KX096705, KX096703, KX096700, KX096704 and KX096702) were isolated in 2016 on the territory of the Turkestan region from ticks *Hyalomma excavatum*, *Hyalomma scupense* and *Hyalomma anatolicum*.

## 3. Discussion

CCHFV remains a globally significant pathogen with serious consequences on human health. The southern regions of Kazakhstan (Zhambyl, Turkestan and Kyzylorda regions) are classified as endemic to CCHF. The surveillance conducted by the laboratory service of Kazakhstan indicates that natural foci are gradually expanding their borders. This is facilitated by the movement of wild and domestic animals in southern regions, as well as the presence of migratory routes of migratory birds passing through the territory of these regions.

Currently, 11 species of ixodes and 1 species of argas ticks have been registered in the southern regions of Kazakhstan [22]. Parasitization of such species as *Hyalomma asiaticum*, *Hyalomma anatolicum*, *Hyalomma marginatum*, *Hyalomma scupence*, *Haemaphysalis numidian*, and *Rhipicephalus schulzei* was noted on domestic animals in the desert zone. Additionally, *Dermacentor daghestanicus*, *Dermacentor marginatus*, *Hyalomma anatolicum*, *Haemaphysalis punctata* and *Rhipicephalus turanicus* were found in the Syrdarya floodplain.

When conducting these studies in 2021 in southern regions of Kazakhstan, we collected five species of ticks: *Hyalomma marginatum* (*n* = 323) (46.5%), *Hyalomma anatolicum* (*n* = 138) (19.9%), *Hyalomma asiaticum* (*n* = 126) (18.2%), *Ixodes ricinus* (*n* = 27) (3.9%) and *Hyalomma scupense* (*n* = 80) (11.5%). It should be noted that on the territory of the Kyzylorda region, we collected 27 *Ixodes ricinus* ticks that are not typical for this area. For the first time, the inhabitation of the *Ixodes ricinus* tick on the territory of the Kyzylorda region was shown by the National Scientific [23]. *Ixodes ricinus* is a new species for the territory of the Kyzylorda region and the territory of Kazakhstan. Therefore, it is most likely introduced with migratory birds or migrating mammals, which requires more detailed research. Due to the small number, the role of this species in the epidemiology of natural-focal infections has not been sufficiently studied.

CCHF has been monitored in Kazakhstan since 2005. According to monitoring data, in Zhambyl, Kyzylorda and the South Kazakhstan regions, the established prevalence of ticks *Hyalomma asiaticum*, *Hyalomma anatolicum* and *Dermacentor daghestanicus* was between 0.3 and 9.0%. In our studies, we found the CCHFV in all three studied areas, which confirms the previously obtained results about the circulation of this virus in these regions. The results of PCR studies of collected ticks showed that the infection rate of ticks *Hyalomma asiaticum* ticks is 2.4% and *Ixodes ricinus* is 3.5%. CCHFV was not detected in the other three tick species studied. We detected the CCHFV in *Ixodes ricinus* tick for the first time. Infection of the *Ixodes ricinus* tick with CCHFV gives reason to believe that this type of tick can participate in the formation and maintenance of a natural focus of CCHF in the south of Kazakhstan. We first discovered the CCHFV in tick *Ixodes ricinus*. Infection of the tick *Ixodes ricinus* with the CCHFV gives rise to the possibility that this type of tick may manifest itself in the disease and the maintenance of an acute focus of CCHF in southern Kazakhstan in the future. To confirm this, additional studies on the infection of this tick species with CCHFV on a larger number of samples and over a longer period of time are needed.

Genetic variants of the CCHFV circulating in studied areas also aroused interest. CCHFV population in Kazakhstan has not been fully characterized, and there is no information about the genetic features of virus isolates. Earlier, it was shown that the genetic variants Asia 1 and Asia 2 are circulating in the Turkestan region, as well as Asia 2 subtype virus, which is circulating in the Kyzylorda region [20,21]. There were no data on the genetic diversity of the CCHFV in the Zhambyl region in the available literature. Our genetic research of the CCHFV S segment has shown that the virus isolated in Zhambyl and Turkestan regions belongs to Asia 2 genotype, and the virus isolated in the Kyzylorda region belongs to the Asia 1 genotype.

Comparison of the CCHFV S segment nucleotide sequences of Asia 1 subtype and phylogenetic analysis of Kazakhstan strain showed 96–100% identity. Within the Asia 2 genotype, the identity of the CCHFV S segment nucleotide sequences of the Kazakhstan strains was 94–100%. Circulation of the CCHFV Asia 2 genetic lineage in the territory of the Zhambyl region was shown for the first time. In addition, it was found that along with the CCHFV Asia 2 genetic lineage that was established earlier [21], the Asia-1 virus also circulates in the territory of the Kyzylorda region.

Based on literature and research results, it can be assumed that two genetic variants of CCHFV circulate on the territory of Turkestan and Kyzylorda regions, and only the Asia 2 genotype is circulating in the Zhambyl region. However, due to the small sample, we cannot reliably state that only the Asia 2 genotype circulates in the Zhambyl region. Most likely, both genotypes circulate in the Zhambyl region. The Zhambyl region border runs to the Turkestan region, where there is a constant movement of wild and domestic animals that can contribute to the spread of both genotypes of CCHFV. The circulation of several genetic variants in the region can lead to the formation of reassortant CCHF strains. Further studies of CCHFV strains belonging to these genetic lineages, including an assessment of the geographical distribution of strains of these lineages in Kazakhstan, are required. This will allow us to assess the genetic diversity and spatial distribution of the Asia 1 and Asia 2 genetic lineages.

## 4. Materials and Methods

### 4.1. Tick Sampling

**Figure 2:**
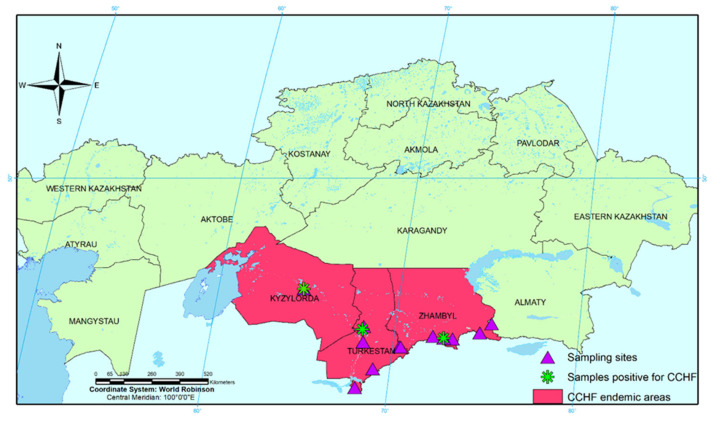
Geographic distribution of CCHF in Kazakhstan.

In 2021, tick samples were collected on the territory of Zhambyl, Turkestan and Kyzylorda regions of the Republic of Kazakhstan (Table 1, Figure 2). A total of 694 tick samples of *Hyalomma marginatum*, *Hyalomma scupense*, *Hyalomma asiaticum*, *Hyalomma anatolicum* and *Ixodes ricinus* were studied, among them 209 ticks from the Zhambyl region, 203 ticks from the Turkestan region and 282 ticks from the Kyzylorda region.

The collection of mature ixodid ticks for research was carried out from vegetation on a flag made of white waffle fabric. Flagging was carried out by performing wave-like movements with a flag (90 × 65 cm) above the surface of plants [24,25]. When counting, the researcher walks at a speed of about 2 km/h, counting steps and dragging the flag next to him through the vegetation. The flag must be constantly unfolded and not rolled into a tube. Tick sampling was carried out at the same time of day (between 09:00 and 12:00 p.m.), regardless of atmospheric conditions, and covering both sunny and shady areas of the 100 m^2^ territory. Inspection of the flag and collection of ticks was carried out every 20 steps. The attached ticks were stored in sterile Eppendorf tubes.

While collecting and accounting for ticks, employees had to follow special precautions such as wearing personal protective clothing with a high neckline and cuffs, also periodic self- and mutual inspections for the presence of ticks.

### 4.2. Transporting Live Ticks

All ticks were sampled alive and placed into plastic test tubes with a screw cap. Usually, a leaf of a cereal plant is thrown into a test tube to maintain humidity. The test tubes were placed in a linen bag and transported in a metal case.

The ticks were delivered for analysis on the day of collection. In the absence of such a possibility, they were kept alive for 10 days in a cool place or in a refrigerator in test tubes with grass. A detailed label was applied to the collected material. Each arthropod was identified using the stereomicroscope RS0745 (Altami, Saint Petersburg, Russia). Species identification of ticks was confirmed morphologically [26,27,28].

### 4.3. Sample Processing and RNA Extraction

After morphological examination, ticks were purified in 70% ethanol, ultra-pure water and again in 70% ethanol, then grounded into a suspension with the addition of PBS (without Ca and Mg ions) [29]. The resulting suspension was stored before the start of the study at −80 °C.

Isolation of viral RNA was performed using the QIAamp Viral RNA Mini Kit (Qiagen GmbH, Hidden) in accordance with the manufacturer’s recommendations from 140 μL of virus-containing fluid. RNA concentrations were measured using a NanoDrop 2000 spectrophotometer (Thermo Fisher Scientific, Waltham, MA, USA). To assess the purity and quality of nucleic acids in spectrophotometric measurement, the purity of the sample was determined based on the ratio of optical densities at A260/A280.

### 4.4. Real-Time Polymerase Chain Reaction

Ticks *Ixodes ricinus*; *Hyalomma marginatum*; *Hyalomma anatolicum*; *Hyalomma asiaticum*; *Hyalomma scupense* collected from Zhambyl, Turkestan and Kyzylorda areas were examined individually.

Real-time PCR was performed using a Rotor-Gene Q thermal cycler (Qiagen, Hilden, Germany). A commercial real-time RT-PCR kit was used for the detection and identification of CCHFV-specific RNA and the causative agent of Q fever (OM-Screen-CCHF/Q-RV, Syntol, Moscow, Russia).

Real-time PCR was performed in a total volume of 35 µL using a MasterCycler ProS cycler (Eppendorf, Hamburg, Germany). The reaction mixture included Reaction PCR Buffer—15.0 μL and template RNA—10 μL.

PCR amplification program: reverse transcription: 15 min 50 °C; denaturation: 5 min 95 °C; number of cycles—50; fluorescence signal reading: 40 s 60 °C and 15 s 95 °C.

### 4.5. PCR for Amplification of CCHFV S Segment

PCR staging was performed using a Qiagen OneStep RT-PCR Kit (Qiagen, Hilden, Germany). RT-PCR was performed in a total volume of 50 µL using a MasterCycler ProS cycler (Eppendorf, Hamburg, Germany). The reaction mixture included: 5× Qiagen OneStep RT-PCR Buffer—10.0 μL, dNTP Mix (10 mM)—2.0 μL, primers 10 pmol each, Qiagen OneStep RT-PCR Enzyme Mix—2.0 μL, template RNA—4 μL.

The primer sequences used in this study: S-rna-CCHF-F1 ACGCCCACAGTGTTCTCTTGAGTG and S-rna-CCHF-R1 CAAGGCCTGTTGCRACAAGTGCTAT) [21]. These primers were synthesized using the DNA/RNA/LNA synthesizer H-16 (K&A Laborgeraete, Schaafheim, Germany). PCR amplification program: reverse transcription step of 50 °C for 30 min; initial PCR activation step of 95 °C for 15 min; 35 cycling of denaturation 94 °C for 0.5 min, annealing 58 °C for 0.5 min, extension 72 °C for 1 min; final extension 72 °C for 10 min.

### 4.6. Sequencing and Phylogenetic Analysis

For sequencing, suspensions of ticks were used, in which RNA was detected by PCR CCHFV. Sequencing was performed on an Applied Biosystems 3130 automated DNA sequencer (HITACHI, Tokyo, Japan) using the BigDye Terminator v3.1 Cycle Sequencing kit (Applied Biosystems, Inc., Vilnius, Lithuania). The resulting nucleotide sequences were analyzed in Sequencer v. 4.5 (“Gene Codes Corporation”, Ann Arbor, MI, USA). The nucleotide sequence was aligned using the computer program complex Mega 7.0. A set of nucleotide sequences from the international GenBank database (NCBI) was used to construct a phylogenetic tree and determine the genotype. The analysis used available nucleotide sequences of CCHFV strains in GenBank (JN572087, JF922680, JX051650, AY297691, AY049083, AY223475, KP075671, KJ676542, KX096701, ON783804, GU477494, FJ562093, DQ227496, DQ227495, KX096703, AJ010648, AY905654, AY297692, AF481799, KX096705, KX096700, ON783805, AY029157, AJ010649, DQ211642, AF362080, DQ446214, AY905660, KP075648, U88414, AY905661, KR075668, KR075655, HM452305, KC867274, AF527810, KX096704, KR075673, CU456723, DQ211645, U15024, KX096702, ON783803, JX908640, AJ538196, KP075667, KR075681, KP075672, KJ485700, U88410, DQ211648, DQ211644, GQ337053, DQ133507, DQ144418, DQ076413, DQ211639, DQ211640 and DQ211638).

The evolutionary history was inferred using the Neighbor-Joining method [30]. The tree is drawn to scale, with branch lengths in the same units as those of the evolutionary distances used to infer the phylogenetic tree. The evolutionary distances were computed using the Maximum Composite Likelihood method [31] and are in the units of the number of base substitutions per site. The proportion of sites where at least 1 unambiguous base is present in at least 1 sequence for each descendent clade is shown next to each internal node in the tree. This analysis involved 59 nucleotide sequences. Evolutionary analyses were conducted in MEGA11 [32].

## Figures and Tables

**Table 1:** Ticks and infections rates for CCHFV in different regions of southern Kazakhstan in 2021.

Ticks	Sample Collection	N Tick	Number of Examined/Number of PCR Positive/Percentage of PCR-Positive Samples			
			Zhambyl Region	Turkestan Region	Kyzylorda Region	Total
*Hyalomma marginatum*	Field	Total	85/0/0	106/0/0	132/0/0	323/0/0
	Field	Females	64/0/0	70/0/0	77/0/0	211/0/0
	Field	Males	21/0/0	36/0/0	55/0/0	112
*Hyalomma scupense*	Vegetation	Total	56/0/0	-	24/0/0	80/0/0
	Vegetation	Females	18/0/0	-	16/0/0	32/0/0
	Vegetation	Males	38/0/0	-	8/0/0	46/0/0
*Hyalomma asiaticum*	Vegetation	Total	50/1/2	34/2/5.9	42/0/0	126/3/2.4
	Vegetation	Females	23/1/4.35	25/2/8.0	13/0/0	61/3/4.9
	Field	Males	27/0/0	9/0/0	29/0/0	65/0/0
*Hyalomma anatolicum*	Vegetation	Total	18/0/0	63/0/0	57/0/0	138/0/0
	Vegetation	Females	7/0/0	39/0/0	22/0/0	68/0/0
	Vegetation	Males	11/0/0	24/0/0	35/0/0	70/0/0
*Ixodes ricinus*	Vegetation	Total	-	-	27/1/3.7	27/1/3.7
	Vegetation	Females	-	-	18/1/5.5	18/1/5.5
	Vegetation	Males	-	-	9/0/0	9/0/0
Total			209/1/0.48	203/2/0.98	282/1/0.35	694/4/0.57

## Data Availability

Not applicable.
